# Testing a General Theory for Optimal Flowering Time in Deciduous Perennial Plants as a Function of Growing Season Length

**DOI:** 10.1111/ele.70315

**Published:** 2026-01-30

**Authors:** John S. Park, John Jackson, Anna Bergsten, Jon Ågren

**Affiliations:** ^1^ Department of Biology University of Oxford Oxford UK; ^2^ Department of Conservation Biology and Global Change Estación Biológica de Doñana (EBD‐CSIC) Sevilla Spain; ^3^ Plant Ecology and Evolution, Department of Ecology and Genetics, Evolutionary Biology Centre Uppsala University Uppsala Sweden

**Keywords:** flowering time, growing season length, life history theory, perennial plants, phenology

## Abstract

Effects of climate change on phenological timing, like flowering onset, are crucial for population fitness and community dynamics. Recent research has focused on plastic responses to earlier springs, but the optimal phenological timing should depend also on the growing season duration, within which entire annual life cycles must unfold. Optimal energy allocation theory can address life‐history scheduling when this critical time window expands. Extending Iwasa and Cohen's (1989) framework, we predict a nonlinear relationship between growing season length and optimal flowering time of deciduous perennial plants measured from spring onset. Common‐garden experiments with purple loosestrife ( *Lythrum salicaria* ) and European goldenrod (*Solidago virgaurea*) along Swedish latitudinal gradients strongly supported this a priori prediction. As climate change alters both start and duration of growing seasons, our finding suggests that optimal flowering time expressed as calendar day could stall before accelerating its advancement in response to climate warming at current high‐latitude range margins.

## Graphical abstract

Climate change affects both the start and duration of growing seasons, creating complex effects on optimal flowering timing that go beyond simple responses to earlier springs. Using optimal energy allocation theory, we found a nonlinear relationship between growing season length and optimal flowering time which was supported by two experiments with perennial species. This nonlinearity reveals inherent limits to phenological advancement under climate warming, with our mathematical approach providing a flexible framework for finding these limits for other species based on their life history.

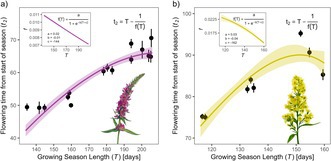

## 1 Introduction

When to reproduce is a critical fitness‐determining decision for seasonal organisms, confined to a limited window of opportunity with conditions favourable for growth: the meteorological growing season (Körner et al. 2023). Climate change is both advancing the onset and prolonging the duration of this window in many systems (Linderholm 2006; Menzel and Fabian 1999). These perturbations have been associated with global changes in biological timing known as phenological shifts (Collins et al. 2021; Parmesan and Yohe 2003; Walther et al. 2002). While in most cases phenology advances, the magnitude and direction of shifts vary considerably among individual organisms, populations and species (CaraDonna et al. 2014; Chmura et al. 2019; Menzel et al. 2006; Miller‐Rushing et al. 2010; Miller‐Rushing and Primack 2008; Prevéy et al. 2019; Thackeray et al. 2010). Importantly, limits to phenological advancement within this limited temporal window under continued climate change are not well understood (Iler et al. 2013; Sparks et al. 2000).

Observed phenology, such as flowering time, results from the complex coordination of many interdependent life‐history processes that include resource acquisition and allocation to growth, reproduction and survival. These processes typically trade off or covary, each with its own time requirements and contribution to fitness (Ehrlén 2015; Ehrlén and Münzbergová 2009; Park and Post 2022; Varpe 2017). Life history theory explains how natural selection for optimal combinations of such processes produces the great diversity of strategies seen in nature (Stearns 1992). While phenology is widely recognised as an outcome of life‐history processes, modern analyses often focus on the shorter‐term plastic responses of isolated phenological traits for which data now abound (Chmura et al. 2019; Cleland and Wolkovich 2024; Kharouba and Wolkovich 2020; Park and Post 2022). An important gap in knowledge is how simultaneous shifts in onset and duration of the season favourable for growth and reproduction affect optimal phenology within the constraints set by life‐history trade‐offs.

Changes in growing season length can require adjustments to annual life‐history schedules to maximise lifetime fitness. Predicting evolutionarily optimal adjustments can be mathematically complex because life‐history processes are bound by invariable temporal order (Post et al. 2008) and trade‐offs can be nonlinear and multidimensional. Perennial organisms must further balance annual scheduling with optimal resource storage for subsequent years to maximise lifetime fitness. Therefore, life history trade‐offs traverse intra‐ and interannual timescales (Cleland and Wolkovich 2024). Yet, most empirical investigations have focused on how system‐specific cues, such as spring onset, proximately induce isolated life cycle events (Chmura et al. 2019; Cleland and Wolkovich 2024). How changes in the length of the growing season—the template within which entire annual life cycles must unfold—shape optimal phenological schedules is far less explored. While the relevant seasonal limits (e.g., temperature or precipitation thresholds) vary by system, limited time is a universal constraint in seasonal systems, offering a more general perspective on phenological adaptation to seasonality.

Optimal energy allocation models provide a direct mathematical translation of the annual life‐history scheduling problem in a general and species agnostic manner (Cohen 1971; Perrin and Sibly 1993). Yet, they have been surprisingly overlooked in the recent surge of empirical work. These models address how an individual should continuously assimilate and expend energy across various life‐history activities to maximise fitness, considering the dynamic consequences of decisions through time. Optimal strategies have been used to analyse population‐ and species‐level differentiation, considering long‐term selection over many generations (Iwasa 2000; Perrin and Sibly 1993). Classic optimal energy allocation models explored broader life‐history divergence across taxa, for example, itero/semelparity (Kozłowski and Wiegert 1986), age at first reproduction (Kozłowski 1992; Kozłowski and Wiegert 1986), and in/determinate growth (Sibly et al. 1985). These classic models typically use the year (age) as the basic unit of time. However, using continuous‐time functions that can represent arbitrarily fine time‐scale processes, a small group of theorists in the 1970s and 80s explored adaptive optimality of *within‐year* life‐history schedules (Cohen 1971; Iwasa 1991, 2000; Johansson et al. 2013; King and Roughgarden 1982a; Kozłowski and Uchmanski 1987). For multi‐year life histories, where growth and stored energy can affect the life cycle in subsequent years, such models use dynamic programming (Perrin and Sibly 1993) to incorporate how a given year's optimal solution is adjusted by its consequences for years later in life.

**FIGURE 1:**
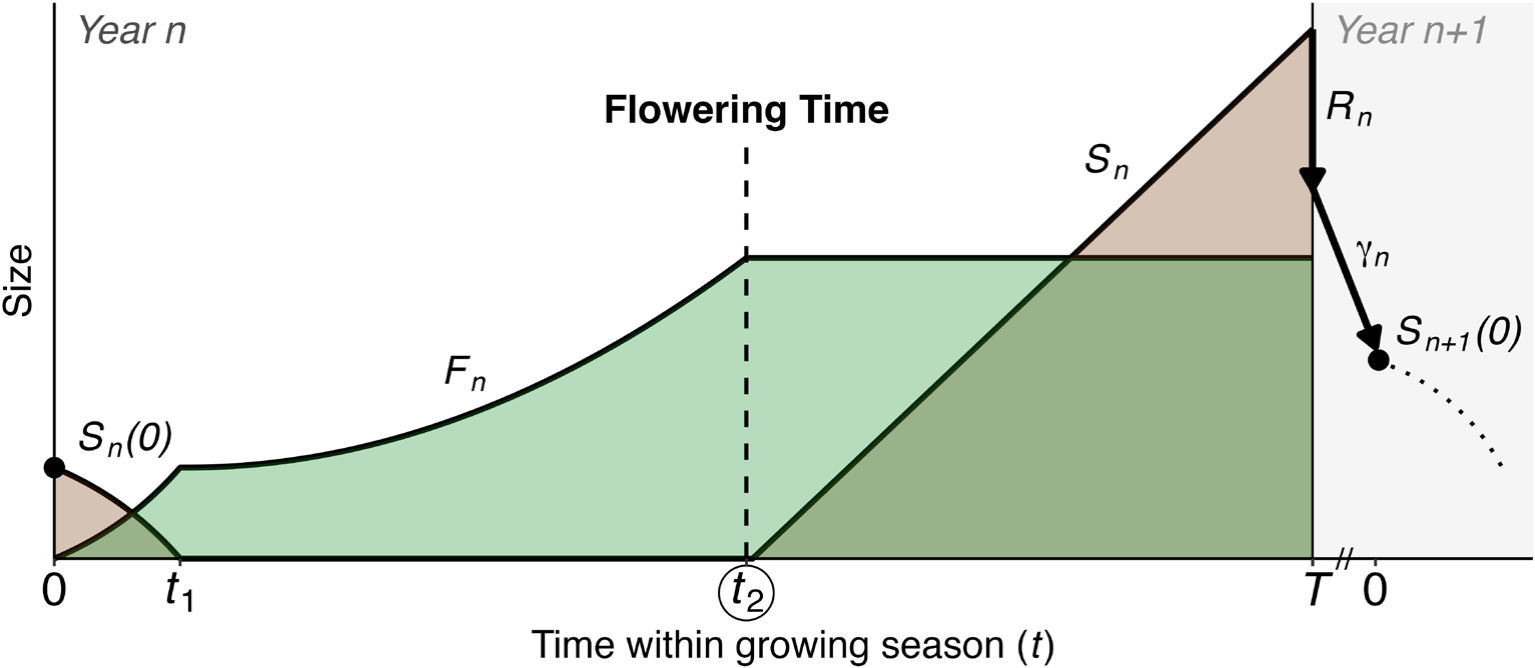
Schematic illustration of the annual schedule of a perennial plant adapted from Iwasa and Cohen (1989). The origin t=0 is the beginning of the growing season, not the year and thus time points along t indicate the number of days relative to the start of the growing season. Two curves with coloured areas show growth functions of the ‘production’ ($F_{n}$) and ‘storage’ ($S_{n}$) parts in year n. ‘Storage’ in the original model includes both actual storage for future use as well as reproductive activity. $F_{n}$ is discarded at the end of growing season T, and $S_{n}\left(T\right)$ is partially allocated to year n's reproductive investment $R_{n}$, and partially lost due to imperfect storage efficiency γ. The storage size to start the following year's life cycle is thus $S_{n+1}\left(0\right)=\gamma \left[S_{n}\left(T\right)-R_{n}\right]$. Discontinuity marks on the *x*‐axis indicate time between growing seasons of year n and year n+1 (i.e., winter). Small t's on the *x*‐axis indicate the two critical switching points, with $t_{2}$ being the beginning of reproductive investment, that is, flowering time, the focus of our study.

In a seminal paper, Iwasa and Cohen (1989) theorised the optimal annual scheduling of vegetative and reproductive activity of deciduous perennial plants. Their work builds on that of Schaffer (1983) and others (Cohen 1971; Iwasa and Roughgarden 1984; King and Roughgarden 1982a; Kozłowski and Wiegert 1986). To introduce what follows, we summarise the original Iwasa‐Cohen model in Figure 1, Box 1 (for a more general review of optimal energy allocation models, see (Iwasa 2000; Perrin and Sibly 1993)).

Here, we examine Iwasa and Cohen's solution for optimal flowering time as a function of growing season length for deciduous iteroparous perennial plants, which start growth each year with resources retained from the preceding year:

(1) $$t_{2}=T-1/f$$

where $t_{2}$ is optimal flowering time counting from the start of the growing season (not the start of the year), T is the length of the growing season and f is the maximum relative growth rate (g/g/day), that is, relative growth rate when the plant is small. Note that this deceptively simple prediction is an outcome of optimising 28 initial parameters that mathematically cancelled out (Iwasa and Cohen 1989). Despite its simplicity and broad implications for phenology, this prediction has gone largely unnoticed and has not been empirically tested to our knowledge.

Relative growth rate f, the mediating parameter, is a key plant life‐history trait (Grime 1977; Stearns 1992; Tilman 1988, 1991) that varies clinally among populations with latitude, thermal regimes and growing season length (Debieu et al. 2013; Grime and Hunt 1975; Li et al. 1998; Mason et al. 2017; McGraw 1985; Montague et al. 2008). Although local factors such as herbivory can impact optimal growth rate (e.g., Colautti et al. 2017), general life history theory predicts that shorter growing seasons should select for increased relative growth rates so that development can be completed within the available time, which has been repeatedly supported empirically (Arendt 1997). Therefore, to test the Iwasa‐Cohen prediction for optimal perennial flowering time across a range of growing season lengths, we must adjust Equation 1 to:

(2) $$t_{2}=T-1/f\left(T\right)$$

**FIGURE 2:**
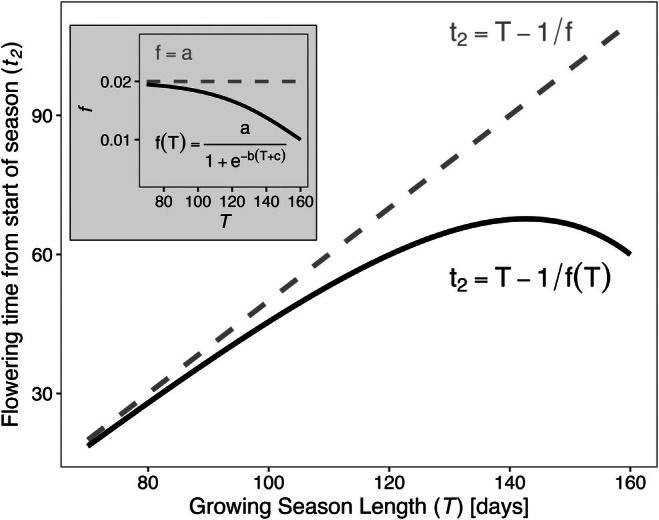
Modification of the Iwasa‐Cohen solution (dashed lines) to incorporate clinal variation in maximum relative growth rate (solid lines), illustrating the nonlinear change in flowering time, expressed as time since beginning of the growing season. Inset shows functions for f underlying flowering time ($t_{2}$) in main plot. Parameter values for illustration: *a* = 0.02, *b* = −0.04, *c* = −160.

We define $f\left(T\right)$ as a biologically realistic yet flexible logistic function, $f\left(T\right)=a/1+e^{-b\left(T+c\right)}$, to model the relative growth rate's dependence on growing season length. The three parameters—a (upper bound), b (steepness) and c (horizontal shift)—allow diverse shapes, including constancy $\left(b=0\right)$ or declines ranging from linear to strongly curvilinear, which also depend on the range of f. This extension generates a nonlinear $t_{2}\left(T\right)$ curve with an initial phase of steady increase, a slowdown, then a subsequent decline (Figure 2). Crucially, because $f\left(T\right)$ appears in the denominator as $1/f\left(T\right)$ in Equation 2, any decline in $f\left(T\right)$, even linear, should induce a nonlinear shape in $t_{2}\left(T\right)$. The nonlinear shape of $t_{2}\left(T\right)$ has two important biological implications. First, it suggests a potential shift in the rate of change in optimal flowering time, measured from the start of the meteorological growing season, as growing season duration expands. Second, if two populations or species occur on different realms of T, or have different functions of $f\left(T\right)$, their changes in optimal flowering time can be quite different given the same absolute change in T.

To test whether the model predicts population differentiation in flowering time across climatic gradients, we leverage the natural gradient of growing season length across latitudes (Appendix Figure S1). We used data from two independent common‐garden experiments with plants sourced from populations across broad latitudinal (57.4°–68.4°) gradients in Sweden. The first comprised 15 populations of the perennial herb *Lythrum salicaria* (Olsson and Ågren 2002). The second comprised 8 boreal populations of the perennial herb *Solidago virgaurea*. We used historical (1961‐) daily weather data to calculate the mean T at each source site. Then, we tested whether flowering time (measured as time since beginning of the growing season) varies following the predicted curvature (Figure 2), given each source population's growing season length and a fitted variation in f across populations. Finally, we calculated expected optimal flowering time expressed as calendar day in situ, by combining model prediction with the mean start of the growing season at the source sites. Using a space‐for‐time substitution, this concurrent effect of earlier springs and longer seasons also predicts how optimal flowering date should change under climate change at a single site.

## 2 Methods

### 2.1 Optimal Flowering Time

We base our study on the optimal scheduling model of deciduous perennial plants initially developed by Iwasa and Cohen (1989) (Box 1). With 28 (life‐history and environmental) parameters, the original model can be tailored to explore several representative classes of plant life histories. We focus on a major class that also mirrors our empirical test systems, namely plants that: (1) grow at the beginning of each season using reserves from the preceding year as well as current‐year photosynthesis, and (2) exhibit non‐instantaneous re‐growth of photosynthetic parts at the onset of each season. Briefly, the model breaks down each year into three phases, highlighting key moments when a plant should switch from using stored resources to fully relying on current photosynthetic growth ($t_{1}$), and then to investing in reproduction and storage ($t_{2}$), with the latter marking the predicted earliest timing of first flower. Given the length of the growing season experienced each year, a combination of yearly and between‐year optimizations determines the solutions for the two critical switching times. Note that solutions pertain to strategies that will be selected for in the long run given they are available in the population's standing variation or via mutation, but the model does not predict reproductive performance year‐to‐year due to plastic adjustments.

BOX 1 **Summary of the Optimal Energy Allocation Model for Perennial Flowering Time.**

*Model overview*

An individual perennial plant consists of a ‘production’ part $\left(F\right)$ and a ‘storage’ part $\left(S\right)$. The former comprises vegetative organs that drive energy generation and growth, that is, leaves, stems and roots, but not trunks or larger branches. The latter encompasses resources stored for use in the following season, as well as allocation to reproductive organs (i.e., flowers and fruits). The objective of the model is to optimise the scheduling of energy allocation to maximise lifetime reproductive investment. Scheduling decisions must balance maximising each year's reproduction with preserving an optimal amount of resources for future reproduction–thus, the problem becomes one of dynamic optimization.

*Summary of the dynamics and optimization*

Daily net production rate depends on the size of F, and drives the growth of F itself as well as S. This production rate follows the function $g\left(F\right)=\mathrm{fF}/\left(1+\mathrm{hF}\right)$, where f is the plant's inherent maximum relative growth rate, which is realised when the plant is small and h is a self‐limiting coefficient.

While the original theoretical framework explores a spectrum of life history scenarios, from here we specifically trace derivations that reflect both our empirical systems and a typical perennial life history. Namely, there are three distinct phases across time t in year n, bounded by growing season length T (Figure 1): (1) $F_{n}$ grows by using both storage $S_{n}\left(t\right)$ and current production $g\left[F_{n}\left(t\right)\right]$. Given conservation of material, $dF_{n}/\mathrm{dt}=g\left[F_{n}\left(t\right)\right]-dS_{n}/\mathrm{dt}$. (2) Storage is depleted, marking the first critical switching point $t_{1}$. From this point, $F_{n}$ continues to grow using production at a rate bounded by $0\leq dF_{n}/\mathrm{dt}\leq aF_{n}+b$, where a and b are limiting constants. (3) The second critical switching point $t_{2}$ occurs when vegetative growth stops, and storage growth $dS_{n}/\mathrm{dt}$ begins.

Both switching points follow ‘bang‐bang control’, meaning an immediate as opposed to a gradual switch, which is a canonical result of evolutionary optimal theory supported by many empirical studies (King and Roughgarden 1982b; Paltridge and Denholm 1974; Perrin and Sibly 1993; Schaffer 1983). While the model does not distinguish between the onsets of flowering, fruiting or storage *sensu stricto* within the third phase, $t_{2}$ marks the end of vegetative growth and the beginning of reproductive investment. Thus we treat $t_{2}$ as flowering time (*cf*. (Johansson et al. 2013; King and Roughgarden 1983)).

At the end of the growing season t=T, any remaining $F_{n}$ is discarded (e.g., leaf shedding), to be reconstructed from storage in the next season. In the beginning of growing season n+1, the size of the storage is $S_{n+1}\left(0\right)=\gamma \left[S_{n}\left(T\right)-R_{n}\right]$, where γ is storage efficiency and $R_{n}$ is reproductive investment in year n.

The overall objective is to maximise lifetime reproductive investment ϕ over n years, that is, find the strategy in which $\phi =\sum_{n=1}^{\infty }\sigma^{n}R^{n}\to \text{maximum}$, where σ is annual survival. This dynamic problem is necessarily divided into ‘within‐season’ and ‘between‐season’ subproblems, where the solution to the former informs the latter as in a mathematical recursion. The first is solved using Pontryagin's Maximum Principle from optimal control theory. Here the variable $\mu \left(t\right)\left(0\leq t\leq T\right)$ is the focal control variable to be optimised, which is the ratio of the growth rate of the production part to its maximum, such that $dF_{n}/\mathrm{dt}=\mu \left(t\right)\left(\mathrm{aF}+b\right)$. The solution involves maximising the Hamiltonian $H\left(F,S,\lambda_{F},\lambda_{S},\mu \right)=\lambda_{F}\mu \left(\mathrm{aF}+b\right)+\lambda_{S}\left[g\left(F\right)-\mu \left(\mathrm{aF}+b\right)\right]$, where $\lambda_{F},\lambda_{S}$ are co‐state variables of F and S. In a simple sense, to find the optimal $t_{2}$ is to find when $\mu \left(t\right)$ should first equal 0. The second problem of optimally dividing each year's $S_{n}\left(T\right)$ into $R_{n}$ and storage, which dictates the optimal schedule for year n+1 and so on, is solved using another mathematical technique called dynamic programming.

The solution of the combined subproblems that we explore in this study is the optimal flowering time as a function of growing season length, which is $t_{2}=T-1/f$. This solution is notably independent of the plant's age (n) or size. We remark that f itself has been found to be related to T in many systems. This modifies the solution to $t_{2}=T-1/f\left(T\right)$, giving a curvilinear function (Figure 2) as opposed to linear, whose downward curvature accelerates as T increases.

To examine whether the model, based on differences in duration of the growing season, can predict population differentiation in flowering time across climatic gradients, we quantified genetically based variation in flowering time among natural populations of *Lythrum salicaria* and *Solidago virgaurea* in common‐garden experiments. Additionally, we assume that by sampling across many source populations (15 for *Lythrum* and 8 for *Solidago*), differences in ecological interactions in situ, such as density‐dependence or herbivory, are minimised as random noise, allowing us to detect signals of the more basal, internal life history optimization pressures imposed by climate seasonality.

### 2.2 Growing Season Lengths, and Start of Spring in Source Populations

The meteorological growing season is defined as the duration within the year in which plant growth can theoretically take place (Carter 1998; Linderholm 2006). Species vary in the relevant thermal conditions that bound their growing seasons. However, a conventional metric widely deemed physiologically favourable for perennial plants, especially in mid‐ to high‐latitudes in the Northern Hemisphere, is when the threshold of 5°C daily mean air temperature (> 5°C for start of spring and < 5°C for start of autumn) for a sustained period (≥ 5 consecutive days) is crossed (Carter 1998; Frich et al. 2002; Körner et al. 2023; Linderholm 2006; Linderholm et al. 2008; Walther and Linderholm 2006). We obtained historical daily mean air temperature data for each source population's site from the high‐resolution gridded PTHBV database generated by the Swedish Meteorological and Hydrological Institute (www.smhi.se). The obtained data spanned from 1961 to years when plants were collected for the respective common‐garden experiments (1997 for *Lythrum*, and 2003 for *Solidago* populations). We calculated the start and end of the growing season for each population in each year, with the difference representing the length of the growing season and subsequently calculated the average growing season length for each population across its year range.

### 2.3 *Lythrum salicaria* Common‐Garden Experiment

Purple loosestrife, *Lythrum salicaria* , is a perennial herb native to Eurasia that has been introduced widely to North America and Australia (Hultén and Fries 1986). In Europe, it is found across the Mediterranean to northern Fennoscandia (36° N–67° N). It occurs in a variety of wetland, lake‐ and seashore, riparian and fen habitats. Common‐garden experiments have demonstrated latitudinal clines in flowering time both in the native range (Olsson and Ågren 2002) and in the introduced range in North America (Colautti et al. 2010). We used data collected and published in (Olsson and Ågren 2002), which contains more detailed information about the sampling protocol and experimental design. Fifteen populations across a wide latitudinal gradient of Sweden (57° N–66° N) were chosen, and seeds were collected from a minimum of 200 individuals per population (Appendix Figure S1b). In May 1997, seeds from 28 to 62 randomly chosen maternal plants from each source population were planted in the greenhouse at Umeå University in northern Sweden (63°49′N). The plants were grown under a photoperiod of 16 h, and 18°C‐day and 8°C‐night temperature cycles. In June 1997, the plants were repotted and transferred to the outdoor experimental garden in a fully randomised design (each maternal family represented by a single plant). In 1999, the phenological status was recorded daily and the date that the first flower opened was recorded for each plant. For our analyses, we converted flowering date to a numerical date relative to the start of the growing season.

### 2.4 Solidago Virgaurea Common‐Garden Experiment

The European goldenrod or woundwort, *Solidago virgaurea*, is a perennial herb widely distributed across Europe, northern Africa and Asia (Hultén and Fries 1986). In Sweden, it occurs in forest and meadow vegetation and reaches into the alpine zone of the Scandinavian mountain range. To assess among‐population variation in flowering time, and the association between flowering start and length of the growing season at the site of origin, we conducted a common‐garden experiment in the Botanical Garden of Uppsala University (59°50′ N). For the present analysis, we used data on flowering time of eight boreal populations located along a latitudinal gradient in Sweden (61° N–68°N; Appendix Figure S1b). Seeds for the experiment were collected between late August and late September 2003. About 2 months after collection, we planted seeds in 11 × 11 × 12 cm plastic pots in an 80:20 mixture of commercial potting soil (‘Weibulls yrkesplantjord’, Weibulls, Hammenhög, Sweden) and LECA (Light Expanded Clay Aggregate, 2–6 mm in diameter, AB Svensk Leca, Linköping, Sweden). Pots were arranged in a randomised block design with 25 blocks. In each block, each population was represented by one plant from a different maternal family. We planted five seeds per pot and emerging plants were thinned to one plant per pot the following spring. In 2005, the great majority of plants in the experiment had reached the flowering stage (median [range], 96% [88%–100%]; *N* = 8 populations). We monitored flowering twice a week once the first flower buds were observed, and for each plant the first day of flowering was recorded. Prior to analysis, we converted flowering date to a numerical date relative to the start of the growing season.

### 2.5 Statistical Analyses

We examined statistical support for the modified, nonlinear Iwasa‐Cohen model using non‐linear Bayesian regression models fit to both population mean and individual flowering times, and compared them to models assuming constant relative growth rate, f, using cross validation. Our non‐linear models included a functional relationship between f and growing season length T ($f\left(T\right)$), as well as the optimised relationship between flowering time ($t_{2}$) and T (Equation 2). We specified a flexible logistic function for $f\left(T\right)$ with three shape parameters: $f\left(T\right)=a/1+e^{-b\left(T+c\right)}$ (Figure 2, inset). We then fitted the function $t_{2}=\left(T-\frac{1}{f\left(T\right)}\right)$ to the data. In constant f models, we explored the linear (slope constrained to 1), intercept‐only relationship between $t_{2}$ and T. We fitted all models in a Bayesian framework using the *brms* package of R (R version 4.3.2 (Bürkner 2017)). We assumed a Gaussian distribution of $t_{2}$ (Appendix Text S1). We ran models across four chains with 4000 iterations (2000 warm‐up), assessing convergence using $\hat{R}$ values. For constant f models, we used weakly informative normal priors. For non‐linear models, we used highly regularised, bounded normal priors to prevent fitting errors due to the asymptotes in the inverse term ($\frac{1}{f\left(T\right)}$). We explored prior bounds using visual prior predictive analysis. For full model structures and prior specifications, see Appendix Text S1. Following model fitting, we compared linear and non‐linear model performance using leave‐one‐out cross‐validation, where each observation is iteratively held out to evaluate model generalizability (*loo* package (Vehtari et al. 2017)). We also used the differences in expected log pointwise predictive density (elpd) to compare model predictive performance. Finally, we assessed parameter uncertainty at the 99% level, and predictions also included population‐level variance σ calculated at the median (50% quantile) level.

## 3 Results

### 3.1 Flowering Time in Both Species Follows the Predicted Nonlinear Relationship to Growing Season Length

**FIGURE 3:**
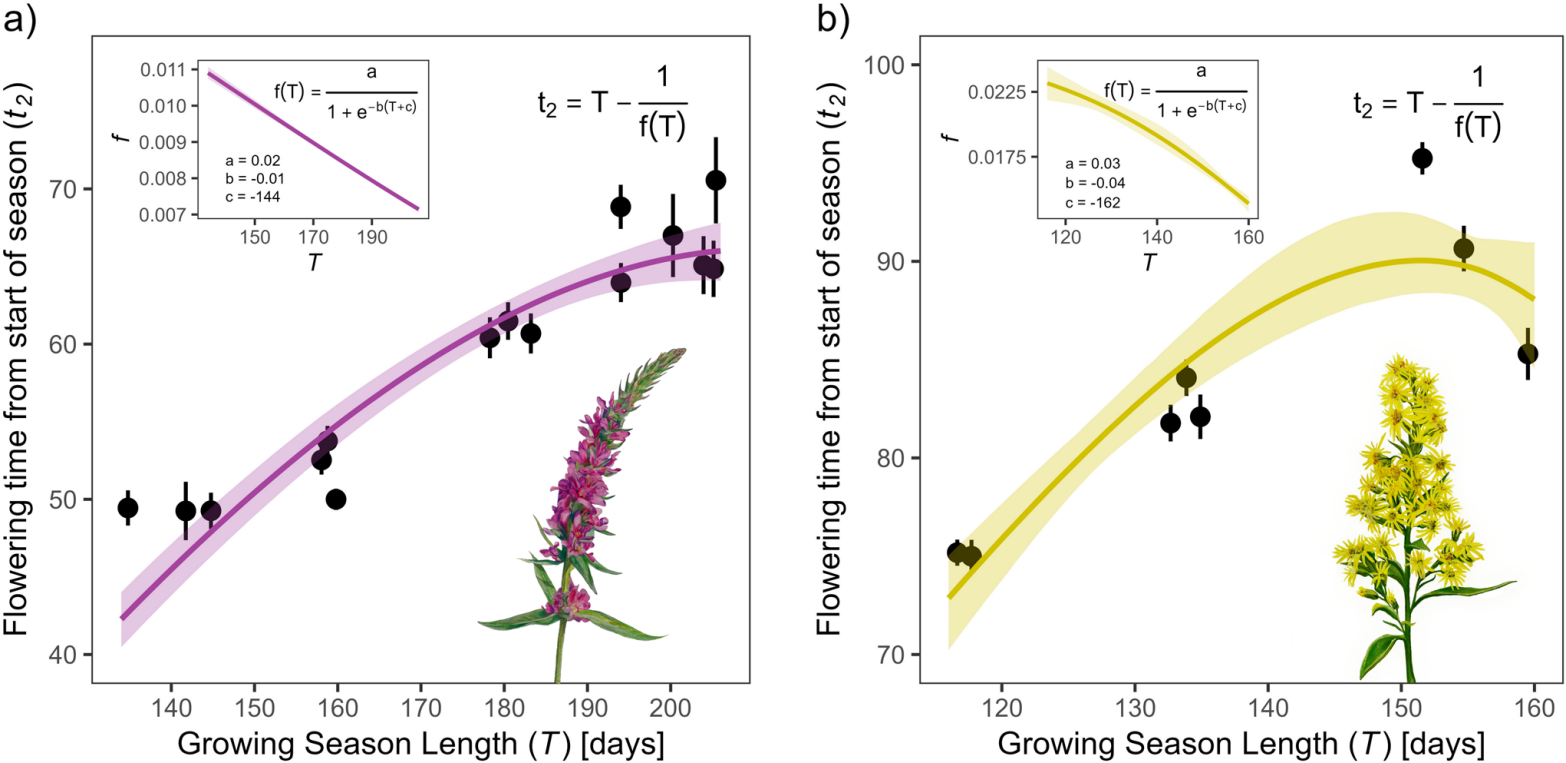
The fitted model of flowering time as a function of growing season length (main plots), and corresponding function of maximum relative growth rate $f\left(T\right)$ (insets), for (a) Lythrum and (b) Solidago common‐garden experiment data. Points are population means of flowering time (days since start of spring), and bars are ±SE. Shaded ribbons in both insets and main plots indicate posterior credible/prediction intervals at the 99% level. Prediction intervals for $t_{2}$ include parameter uncertainty and posterior mean population level variance in $t_{2}$, σ (Appendix Text S1). Illustrations courtesy of Becca Huggins.

Mean growing season lengths in *Lythrum* source population sites ranged from 134.8 to 205.4 days, and in *Solidago* populations from 116.7 to 159.5 days. In the common‐garden experiments, population means of flowering time spanned 21 days for *Lythrum* (5 July to 27 July, corresponding to 50–71 days after the start of the local growing season, 17 May) and 21 days for *Solidago* (15 June to 6 July, 75–96 days after the start of growing season, 1 April) (full individual data shown in Appendix Figure S1a). Posterior estimates for fitted shape parameters in $t_{2}=T-\frac{1}{a/1+e^{-b\left(T+c\right)}}$ were $a=0.021\;\left[99\%\text{credible interval}:0.018,0.024\right]$, $b=-0.010\;\left[-0.012,-0.009\right]$ and $c=-144\;\left[-168,-120\right]$ across *Lythrum* populations, and $a=0.026\;\left[0.022,0.039\right]$, $b=-0.043\;\left[-0.083,-0.0.023\right]$ and $c=-162\left[-167,-138\right]$ across *Solidago* populations (Figure 3) (Appendix Text S1 for full model structure). In both species, we found strong statistical support for our modified Iwasa‐Cohen model that predicted a specific nonlinear functional relationship between growing season length and optimal flowering time. Using leave‐one‐out cross validation, we found that the modified model substantially increased predictive performance compared to models that assumed no variation in the mediating parameter f across T (Table 1).

**TABLE 1:** Predictive performance and leave‐one‐out information criterion (LOOIC) comparison between the modified, nonlinear Iwasa‐Cohen model ($t_{2}=T-1/f\left(T\right)$) and a model with constant f ($t_{2}=T-1/f$). elpd is the expected log pointwise predictive density, ∆ elpd gives the difference in elpd between models and elpd se indicates the standard error in elpd.

	elpd	∆elpd	elpdse	LOOIC
Lythrum model				
Iwasa‐Cohen model $\left[f\left(T\right)\right]$	−1821.3	0	30.9	3642.6
Constant f	−2172.3	−351	15.8	4344.5
Solidago model				
Iwasa‐Cohen model $\left[f\left(T\right)\right]$	−583.8	0	12.6	1167.6
Constant f	−716.1	−132.3	8.9	1432.2

The increasing curvature of the relationship between length of the growing season and optimal flowering time counting from the start of the season, $t_{2}=T-1/f\left(T\right)$, is caused by f declining in the denominator; as f gets smaller than 1, the subtraction $-1/f\left(T\right)$ increases. The predicted $t_{2}\left(T\right)$ curvature increases even more beyond the range of T we observed across our source sites and mathematically has a vertical asymptote (i.e., infinite drop in $t_{2}$). However, this unrealistic extreme limit is biologically irrelevant because the asymptote occurs when f, maximum relative growth rate, is 0. We expect that the decline of $f\left(T\right)$ will indeed have a biological lower limit, as in the logistic function we fitted. The estimated ranges of f (see insets in Figure 3a,b) roughly corroborate various reports of relative growth rates in the literature ($<0.002\sim 0.26\cdot {\mathrm{day}}^{-1}$ for *Lythrum salicaria* (Mckenna and Shipley 1999; Shipley and Peters 1990; Sit 2021; Willis et al. 1999), and $<0.007\sim 0.12\cdot {\mathrm{day}}^{-1}$ for *Solidago altissima* (Meyer 1993; Meyer and Whitlow 1992; Uriarte 2000), a congeneric species for which reports were available).

### 3.2 Shifts in Spring Onset and Growing Season Length Together Result in Accelerating Advancement in Optimal Flowering Time

**FIGURE 4:**
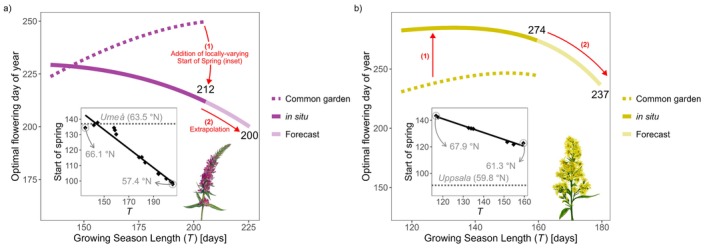
Estimates of in situ optimal flowering time expressed as day of the year across (a) Lythrum and (b) Solidago populations, and predictions beyond the observed range of growing season lengths. Dotted grey line in each inset shows the start of spring at the common‐garden sites of the respective experiments (Umeå for Lythrum, Uppsala for Solidago), in the experiment year. Black points are mean in situ start of spring against growing season length between 1961 and year of plant collection at the source sites (1997 for Lythrum, 2003 for Solidago), and black lines are linear regressions. Dotted coloured curves in the main plots are fitted optimal flowering ($t_{2}$) predictions across source sites in the common‐gardens (flowering time expressed as number of days after the start of the growing season), identical to Figure 3. Coloured solid curves show expected in situ optimal flowering (expressed as day of the year) across *T*, and are summations of the in situ start of spring regression lines and the $t_{2}$ curves. Lighter curves are extrapolations for a further 20‐day increase in T under the fitted functions, with predicted optimal calendar day of flowering labelled. Illustrations courtesy of Becca Huggins.

The function $t_{2}=T-1/f\left(T\right)$ describes the optimal flowering time expressed as days after the start of the growing season, predicting flowering times in common‐garden experiments where all plants experience the same start of the growing season. To translate this into predicted optimal flowering time in the natural populations expressed as calendar day, differences in the start of the growing season need to be considered. Across source sites, the start of the growing season covaries strongly with growing season length (i.e., earlier spring ≈ longer growing season; Appendix Figure S2), and differs from those of the sites where the common‐garden experiments were conducted. Combining the variations in growing season length with the variations in start of spring at the source population sites tilts the $t_{2}$ curve clockwise (Figure 4, step (1) to produce solid coloured curves), to give the expected optimal flowering times expressed as calendar days in situ.

The tilted curves predicting the optimal calendar day of flowering in situ across the latitudinal gradient also predict optimal flowering time in a single locality under climate change, assuming that the start of spring and growing season length change together (as supported by historical data from these sites; Appendix Figure S3).

Finally, we extrapolated the tested function by extending T by 20 days—the average observed increase across sites since 1961 (Appendix Figure S3)—to simulate continued climate change (Keeling et al. 1996; Linderholm 2006; Menzel and Fabian 1999). If the relationship between start of spring and T (Figure 4 insets) remains constant, this predicts rapid nonlinear advancements in optimal flowering time (12 days for *Lythrum* and 37 days for *Solidago*), just beyond the range of growing season lengths observed at the source sites (Figure 4, step (2) to extrapolate). Assuming $f\left(T\right)$ continues to follow the fitted logistic curves, a 20‐day increase in T yields f=0.006 for *Lythrum* and f=0.008 for *Solidago*, values still within biologically realistic ranges from the previous studies. For comparison, applying a linear approximation using the slope of the tilted $t_{2}$ curves at maximum observed T (−0.50 for *Lythrum* and−1.0 for *Solidago*) would predict only a 10‐day advancement for *Lythrum* and 20 days for *Solidago*, underestimating shifts by 2 and 17 days, respectively. Note that these are conservative error estimates compared to applying a linear correlation across the entire observed *T* range.

## 4 Discussion

The growing season is a critical window for all annual life‐history activities. Despite variations among species in specific activities and their impact on lifetime fitness, selection for packing annual scheduling within limited favourable conditions is a unifying process that can help explain geographical variations and climate‐driven shifts in optimal phenology. Using optimal energy allocation theory, we predicted a nonlinear relationship between growing season length and optimal flowering time. This relationship produces a decelerating slope between growing season length and optimal flowering time due to an inverse term involving maximum relative growth rate, a key life‐history trait. Independent common‐garden experiments for two species validated this nonlinear functional shape, using geographic variation in growing season length and genetically based population differentiation in flowering time as a proxy for differences in optimal flowering time.

The nonlinear curves of flowering time advancement carry important implications. First, in a space‐for‐time perspective, populations at higher latitudes—with the shortest growing season (e.g., T≲150 for *Lythrum*; ≲130 for *Solidago*)—show a slow (*Lythrum*) or almost no (*Solidago*) advancement in optimal flowering date as the growing season lengthens. The slope of this change and the duration of relative stasis will vary among species, depending on their specific $f\left(T\right)$ function and the relationship between growing season length and spring onset in their environment (Figure 4, insets). Second, beyond a critical growing season length T, optimal flowering shifts accelerate. The threshold T for this acceleration will depend on the species‐specific $f\left(T\right)$ function but, because f appears in the denominator, such acceleration should emerge in any species as long as f decreases with increasing T. Direct measurements of the lower asymptotic limits of $f\left(T\right)$ will clarify the limits of flowering time advancement beyond the acceleration phase. Testing the model's predictive power in other species will require careful quantification of growing season length and $f\left(T\right)$, which may differ even for sympatric species due to variation in thermal requirements for growth. These predictions apply primarily to deciduous perennials that develop vegetative structures before flowering; thus they may not hold for species that flower first, such as spring ephemerals (Iwasa and Cohen 1989).

While an expansion of the meteorological growing season is a globally reported phenomenon under climate change, the rate and symmetry of that expansion varies geographically (Carter 1998; Kukal and Irmak 2018; Linderholm 2006; Linderholm et al. 2008; Menzel and Fabian 1999; Song et al. 2010). In particular, the contribution of autumn delay to growing season dynamics is much less investigated than that of spring advancement (Gallinat et al. 2015) (but see (Collins et al. 2021; Estrella and Menzel 2006; Gunderson et al. 2012; Zhu et al. 2012)). In some environments, one further complicating feature is the increase in summer dry periods that effectively reduces or breaks up the growing season, even if the boundaries of the season defined by temperature thresholds might be expanding (Cook and Vizy 2012; Iler et al. 2019; Padrón et al. 2020; Trnka et al. 2011). The impact of increasingly frequent summer droughts on optimal flowering time warrants further study, for instance through integration of how drought stress might affect the maximum relative growth rate on which optimality depends. Optimal energy allocation models are well suited for such questions. Importantly, the precise climatic boundaries relevant for modelling are system‐specific; the 5‐day $5^{{}^{\circ}}C$ threshold we used to define the growing season is broadly applicable and common, but not universal. Different temperature thresholds or other abiotic factors such as water availability or photoperiod should be applied for other species when known. Synergistic effects of multiple thresholds in determining growing season length and their impact on long‐term flowering time selection would further improve our findings. Particularly, the decoupling of variable (e.g., temperature) and invariable (e.g., photoperiod) factors delimiting the growing season under climate change should lead to more complex but useful extensions to our model which mathematically considers a single, abstract growing season demarcation.

Iwasa and Cohen's optimization solution predicted an outsized role of maximum relative growth rate f in mediating the relationship between growing season length and optimal flowering time. Intriguingly, this corroborates a long‐standing tenet of plant life history theory, that relative growth rate is a comprehensive trait through which many important fitness‐related traits can be explained (Arendt 1997; Grime 1977; Grime and Hunt 1975; Stearns 1992). In our study, f was a free parameter that we fitted. However, f can be inferred from individual growth curves, for example, by fitting a logistic function to growth timeseries and computing the peak of its derivative (rate of acceleration). Which measurable part of the plant would most accurately reflect the f parameter will depend on the species. The clinal variation of f along growing season length gradients will also vary between systems. Here we chose a logistic function that is biologically realistic and flexible. The shape parameters that govern the slope and curvature of the function $f\left(T\right)=a/1+e^{-b\left(T+c\right)}$ have strong influences on the resulting curvature of $t_{2}=T-1/f\left(T\right)$. While f might indeed be a conserved trait in some species, we posit this would be an exception rather than the rule, as growth rate is a well‐established axis of trade‐off with other canonical life‐history traits (Arendt 1997; Grime 1977; Grime and Hunt 1975; Stearns 1992). Yet, note that our fitting routine allowed f to be conserved across T (when b=0). For a given species, any decrease in f with growing season length would mathematically cause a nonlinear relationship between growing season length and optimal flowering time.

Common‐garden experiments are ideal for our validation because they minimise the impact of unmeasured in situ environmental differences. However, several microhabitat features or ecological interactions can alter the trajectory of evolution in situ from what we have predicted based on climatic selection on optimal phenology. For instance, by incurring extra cost functions, herbivory (Lehndal and Ågren 2015) and density‐dependence (Takada and Nakajima 1996) might alter the control function $\mu \left(t\right)\left(0\leq t\leq T\right)$, that is, how the ratio of the growth of the vegetative production part to its maximum rate changes through time (Box 1). In other words, herbivory and density‐dependence may dampen the growth of the production part of the plant, and possibly require additional maintenance costs, during the part of the season when they are applicable. As all solutions of the optimization directly or indirectly hinge on this control function, such influences could change the optimal flowering time. Other factors such as interactions with pollinators, seed dispersers or predators may also influence the control function $\mu \left(t\right)$ for a subset of t (Elzinga et al. 2007), and adjust optimal solutions. Beyond altering optimal predictions, variations in such biotic factors and numerous abiotic ones (e.g., soil moisture, photoperiod) can cause plastic variation in phenology. Here, we assumed that population differentiation in flowering time across the sampled climatic gradient was adaptive and thus reflected variation in optimal flowering time, and that factors in addition to T that influence the optimum were reasonably similar or varied unsystematically across sampled populations. In both species independently, we found evidence for considerable genetic differentiation in flowering time between populations that follows our optimality‐based prediction. Further, the duration of the growing season at a given site typically varies among years, which likely produces most of the observed plastic shifts in phenology at ecological timescales. If there is an environmental signal early in the season that the plant can perceive and that predicts the expected duration of the growing season, the model would also predict the optimal plastic response, that is, the optimal reaction norm in relation to this signal.

Determining evolutionarily optimal phenological timing in changing seasonal environments involves two key complexities. First, the life‐history processes that shape an individual's schedule are interconnected through covariances and trade‐offs. The common assumption that earlier springs should lead to earlier phenology oversimplifies the relationship between seasonality and phenology, with limited long‐term predictability. This view also fails to explain the observed variability in phenological shifts (Chmura et al. 2019). In contrast, life history theory emphasizes long‐term selection and fitness optimization beyond immediate plastic responses. To fully treat phenology as a life history phenomenon, we must consider how the interconnected suite of life‐history processes should optimally reschedule in response to changes in seasonal time available for all processes. Second, although the onset and duration of the season are related, they exert distinct albeit synergistic influences on optimal timing. Optimal energy allocation models are well suited to address both complexities in a mathematically explicit and adaptable way. Our extension of the Iwasa‐Cohen hypothesis (Equation 2) predicted a nonlinear relationship between optimal flowering time and growing season length—one that would be obscured under the assumption that phenology primarily tracks the start of spring. We advocate for a priori quantitative hypotheses grounded in life history theory to move beyond *post hoc* explanations, clarifying how seasonal time pressures mechanistically select for timing patterns and enabling systematic predictions across systems.

## Author Contributions

John S. Park and Jon Ågren designed research. Anna Bergsten and Jon Ågren designed the common‐garden experiment and collected data. John Jackson and John S. Park analysed data. John S. Park, John Jackson and Jon Ågren wrote the paper.

## Funding

This work was supported by H2020 Marie Skłodowska‐Curie Actions, 101030973. Swedish Research Council, 2023–05270. HORIZON EUROPE Marie Sklodowska‐Curie Actions, 101067850.

## Conflicts of Interest

The authors declare no conflicts of interest.

## Supporting information


**Data S1:** ele70315‐sup‐0001‐FigureS1.docx.

## Data Availability

The data and R scripts that support the findings of this study are openly available at https://github.com/jjackson‐eco/flowering_time_growing_season_length and https://zenodo.org/records/14045223.
