# Supplementary material for: Testing a General Theory for Optimal Flowering Time in Deciduous Perennial Plants as a Function of Growing Season Length
Source: Ecol Lett. 2026 Jan 30;29(2):e70315. doi: 10.1111/ele.70315 (PMC12858690; doi:10.1111/ele.70315)
Supplement: Supplementary file 1 — Data S1: ele70315‐sup‐0001‐FigureS1.docx. [file ELE-29-0-s001.docx]

**Supporting Information for:**

Testing a general theory for flowering time shift as a function of growing season length

**Authors:**

John S. Park, John Jackson, Anna Bergsten, Jon Ågren

**Corresponding author:** John S. Park

**Email:** *john.park@biology.ox.ac.uk*

**This PDF file includes:**

Figures S1 to S3

Supporting Text S1


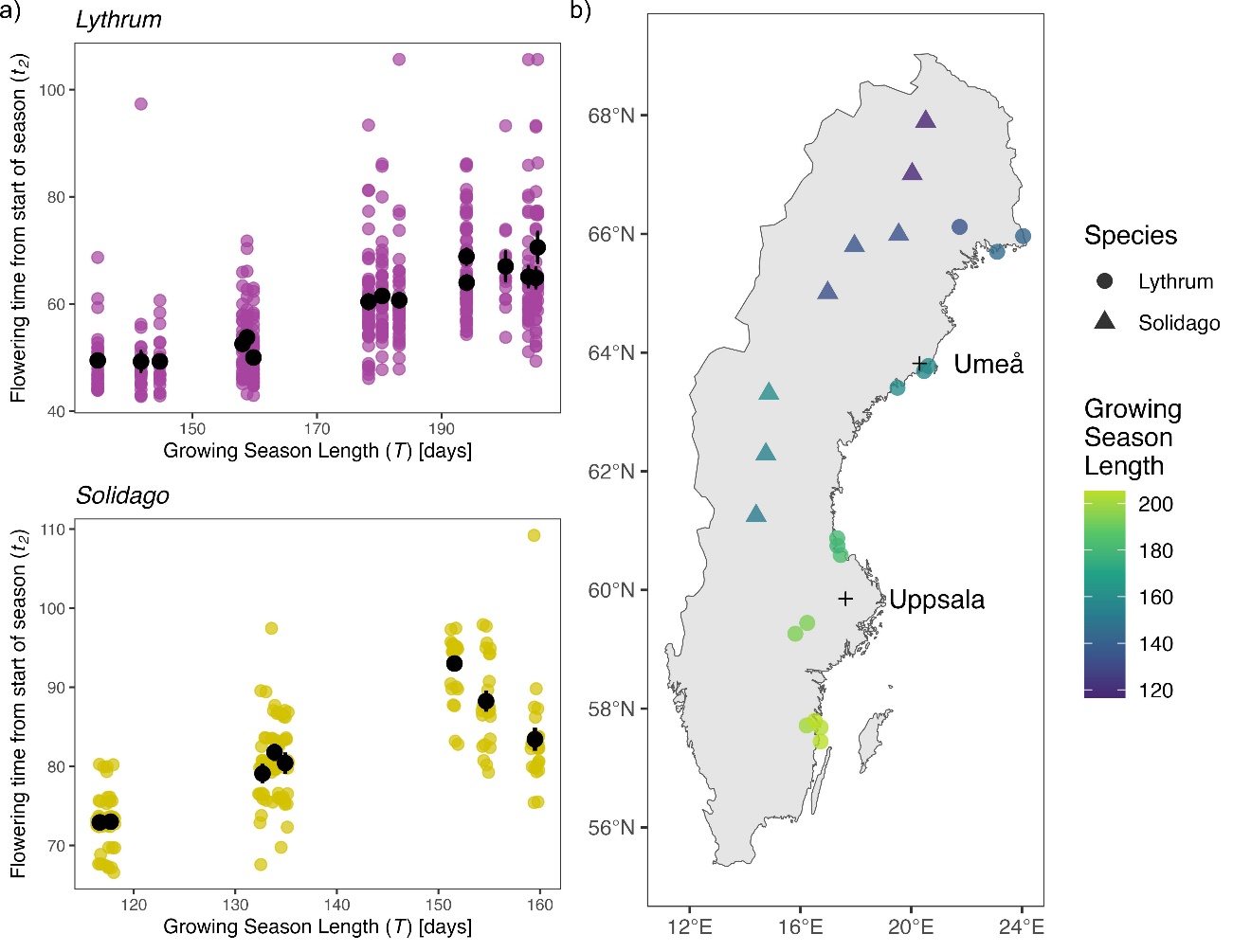


**Figure S1. Flowering time data and source population sites.** a) Colored points show individual plant flowering times (flowering initiation) measured from the start of spring ($t_{2}$) in the respective common garden experiment location in the experiment year (Umeå in 1999 for *Lythrum*, and Uppsala in 2005 for *Solidago),* plotted against growing season length ($T$) of source population site. $T$ is the average growing season length from 1961 to collection year (1997 for *Lythrum* and 2003 for *Solidago*), measured as duration between the first and last time the threshold of 5 consecutive days of 5ºC daily mean temperature is crossed. b) Circle and triangle points show locations of *Lythrum* and *Solidago* source populations, respectively, from which seeds for the common garden experiments were collected. Colors are growing season lengths ($T$) at each locality.

**Supporting Information Text**

Supporting Information Text S1. Full model structure and prior distribution specifications for the Bayesian regression models.

**Constant** $\boldsymbol{f}$ **(original Iwasa-Cohen) model structure (both *Lythrum* and *Solidago*)**

$$t_{2} \sim Normal\left( \mu, \sigma\right)$$

$$\mu_{i}=T_{i}-1/f$$

Priors

$$f \sim Normal\left( 0, 0.1 \right)$$

$\sigma\sim Normal(0, 5)$, for $0<\sigma$

**Extended Iwasa-Cohen (variable** $\boldsymbol{f}$**) model structure: *Lythrum***

$$t_{2} \sim Normal\left( \mu, \sigma\right)$$

$$\mu_{i}=T_{i}-\frac{1}{a/{1+e^{-b(T+c)}}}$$

Priors

$a \sim Normal\left( 0.05, 1 \right)$, for $0.0001<a<1$

$b \sim Normal\left( -0.05, 1 \right)$, for $-1<b<-0.0001$

$c \sim Normal\left( -150, 10 \right)$, for $-200<c<-100$

$\sigma\sim Normal(0, 5)$, for $0<\sigma$

**Extended Iwasa-Cohen (variable** $\boldsymbol{f}$**) model structure: *Solidago***

$$t_{2} \sim Normal\left( \mu,\sigma\right)$$

$$\mu_{i}=T_{i}-\frac{1}{a/{1+e^{-b(T+c)}}}$$

Priors

$a \sim Normal\left( 0.5, 0.75 \right)$, for $0.0001<a<1$

$b \sim Normal\left( -0.5, 0.75 \right)$, for $-1<b<-0.0001$

$c \sim Normal\left( -150, 10 \right)$, for $-200<c<-100$

$\sigma\sim Normal(0, 5)$, for $0<\sigma$

**
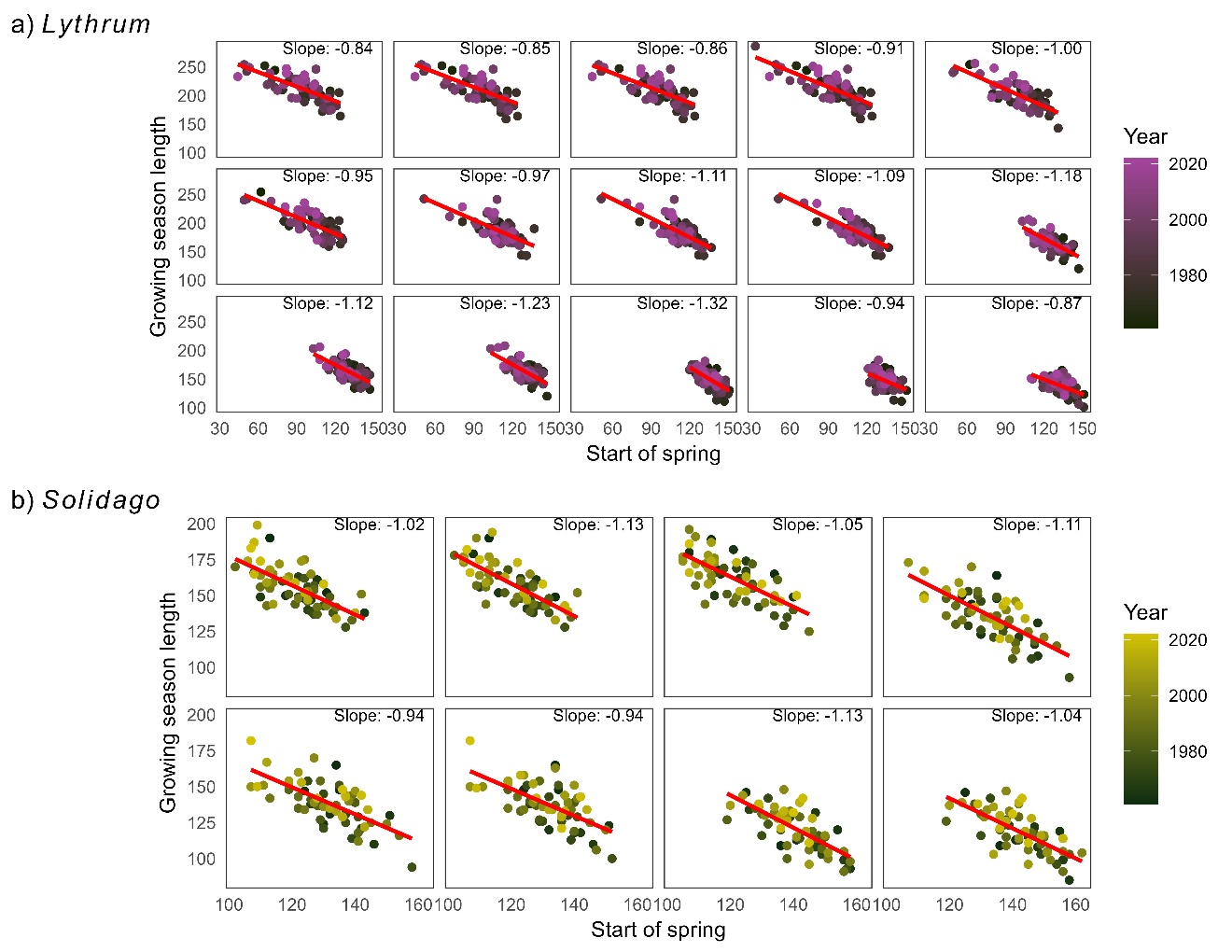
Figure S2. Association between growing season length and start of spring at a) *Lythrum* and b) *Solidago* source sites from 1961 to 2022.** Each panel represents a source site, with colored points corresponding to year. Lines and slope values show linear regression in each site. Panels are organized in increasing latitude of source site, from left to right and top to bottom.


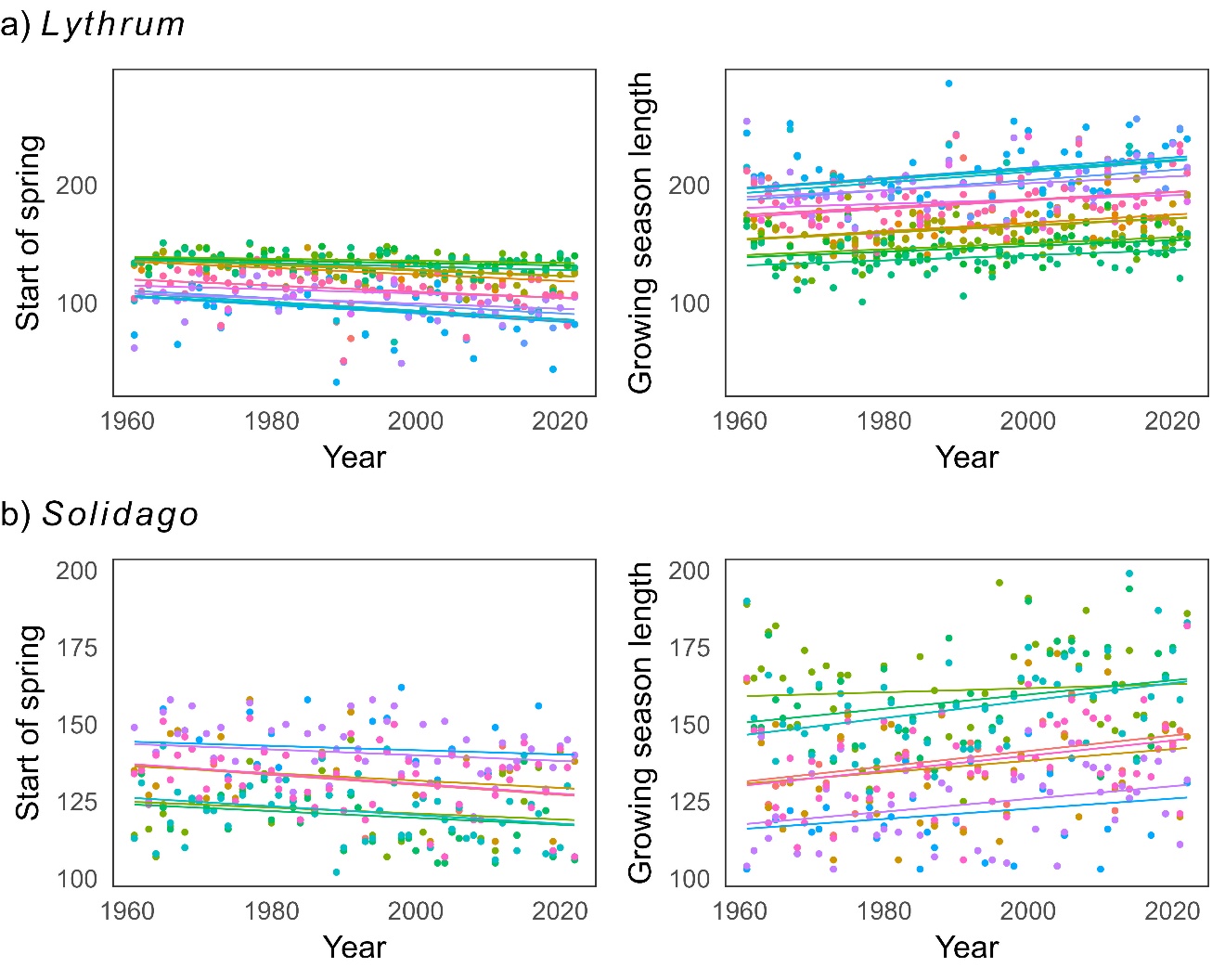


**Figure S3. Historical trends in start of spring and growing season length in a) *Lythrum* and b) *Solidago* source sites.** Colors correspond to source sites (15 for *Lythrum* and 8 for *Solidago*), with points being metrics per year and lines being linear regressions through them.
